# Concurrent IDH1 and IDH2 mutations in glioblastoma: A case report

**DOI:** 10.3389/fonc.2023.1071792

**Published:** 2023-04-03

**Authors:** Ali S. Haider, Chibawanye I. Ene, Paolo Palmisciano, Maryam Haider, Ganesh Rao, Leomar Y. Ballester, Gregory N. Fuller

**Affiliations:** ^1^Department of Neurosurgery, The University of Texas M.D. Anderson Cancer Center, Houston, TX, United States; ^2^Department of Neurosurgery, University of Cincinnati College of Medicine, Cincinnati, OH, United States; ^3^Department of Radiology, Baylor College of Medicine, Houston, TX, United States; ^4^Department of Neurosurgery, Baylor College of Medicine, Houston, TX, United States; ^5^Department of Pathology, The University of Texas M.D. Anderson Cancer Center, Houston, TX, United States

**Keywords:** glioblastoma, histopathology, isocitrate dehydrogenase, somatic mutation, tumor progression

## Abstract

Isocitrate dehydrogenase (IDH) mutations are cornerstone diagnostic features in glioma classification. IDH mutations are typically characterized by mutually exclusive amino acid substitutions in the genes encoding for the *IDH1* and the *IDH2* enzyme isoforms. We report our institutional case of a diffuse astrocytoma with progression to secondary glioblastoma and concurrent IDH1/IDH2 mutations. A 49-year-old male underwent a subtotal resection of a lobular lesion within the right insula in 2013, revealing a WHO grade 3 anaplastic oligoastrocytoma, IDH1 mutated, 1p19q intact. Symptomatic tumor progression was suspected in 2018, leading to a surgical tumor biopsy that demonstrated WHO grade 4 *IDH1* and *IDH2* mutant diffuse astrocytoma. The patient subsequently underwent surgical resection followed by medical management and finally died in 2021. Although concurrent *IDH1/IDH2* mutations have been rarely reported in the current literature, further study is required to better define their impact on patients’ prognoses and their response to targeted therapies.

## 1 Introduction

Isocitrate dehydrogenase (IDH) mutations were first identified in diffuse gliomas in 2009 and have since played a crucial role in glioma classification (1, 2). The 2016 World Health Organization (WHO) Classification of Tumors of the Central Nervous System incorporated IDH classification as a cornerstone diagnostic feature, which has continued in the 2021 update (3, 4). IDH mutations typically result in mutually exclusive amino acid substitutions in genes encoding for the *IDH1* (cytoplasmic isoform) or *IDH2* (mitochondrial isoform) enzyme (5). Histopathological confirmation, IDH mutation status, and 1p/19q codeletion status allows the molecular stratification of diffuse gliomas into 3 subgroups with significant survival implications: 1) IDH mutant, 1p/19q codeleted oligodendrogliomas; 2) IDH mutant, 1p/19q non-codeleted astrocytoma; 3) IDH wildtype gliomas (3, 6, 7). Though *IDH1* and *IDH2* mutations are usually mutually exclusive, we report a case seen at our institution of diffuse astrocytoma with progression to secondary glioblastoma and concurrent IDH1/IDH2 mutations.

### 2 Case presentation

**Figure 1:**
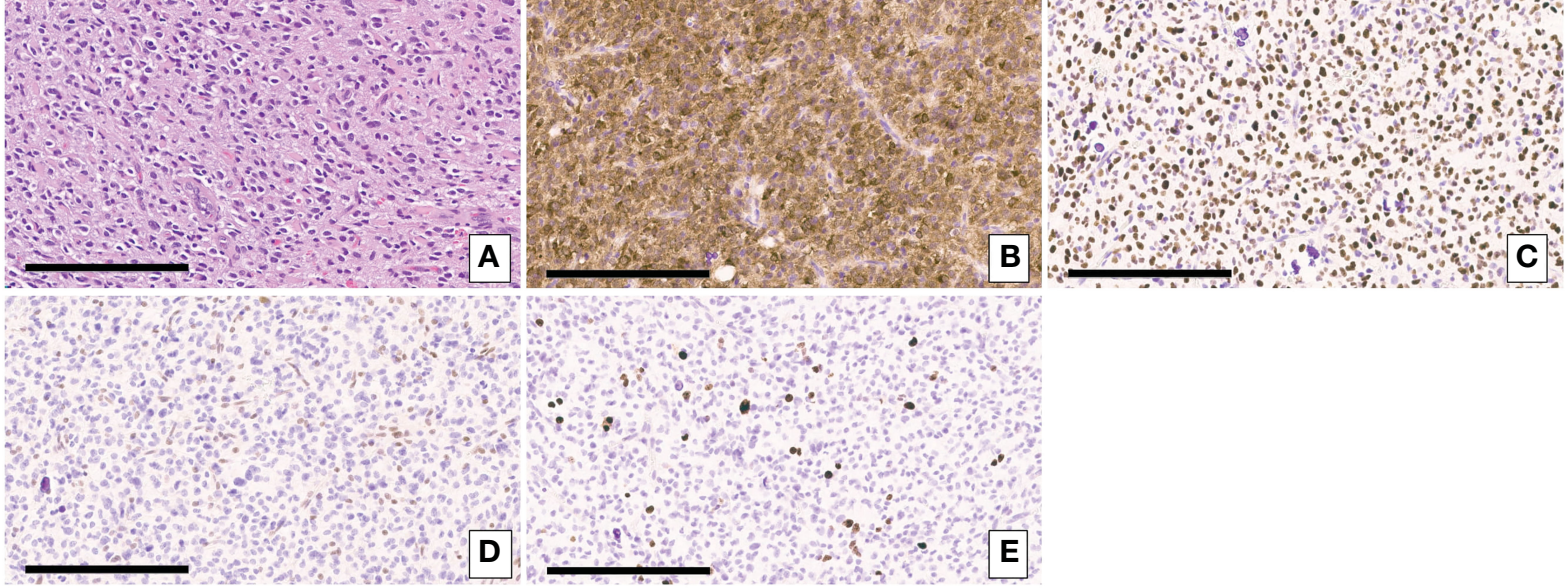
**(A)** H&E-stained sections of the biopsy showing a densely cellular diffuse glioma with vascular proliferation composed predominantly of cells with relatively uniform round nuclei, many of which had perinuclear halos. **(B)** Immunohistochemical staining for IDH1 p.R132H showing expression of the mutant protein in tumor cells. **(C)** Immunohistochemical staining for p53 showing strong nuclear staining in the majority of tumor cells consistent with the presence of a *TP53* mutation. **(D)** Immunohistochemical staining for ATRX demonstrating loss of ATRX expression tumor cells but retained normal ATRX expression in endothelial cells. **(E)** Immunohistochemical staining for Ki67. Scale bar = 200um.

**Figure 2:**
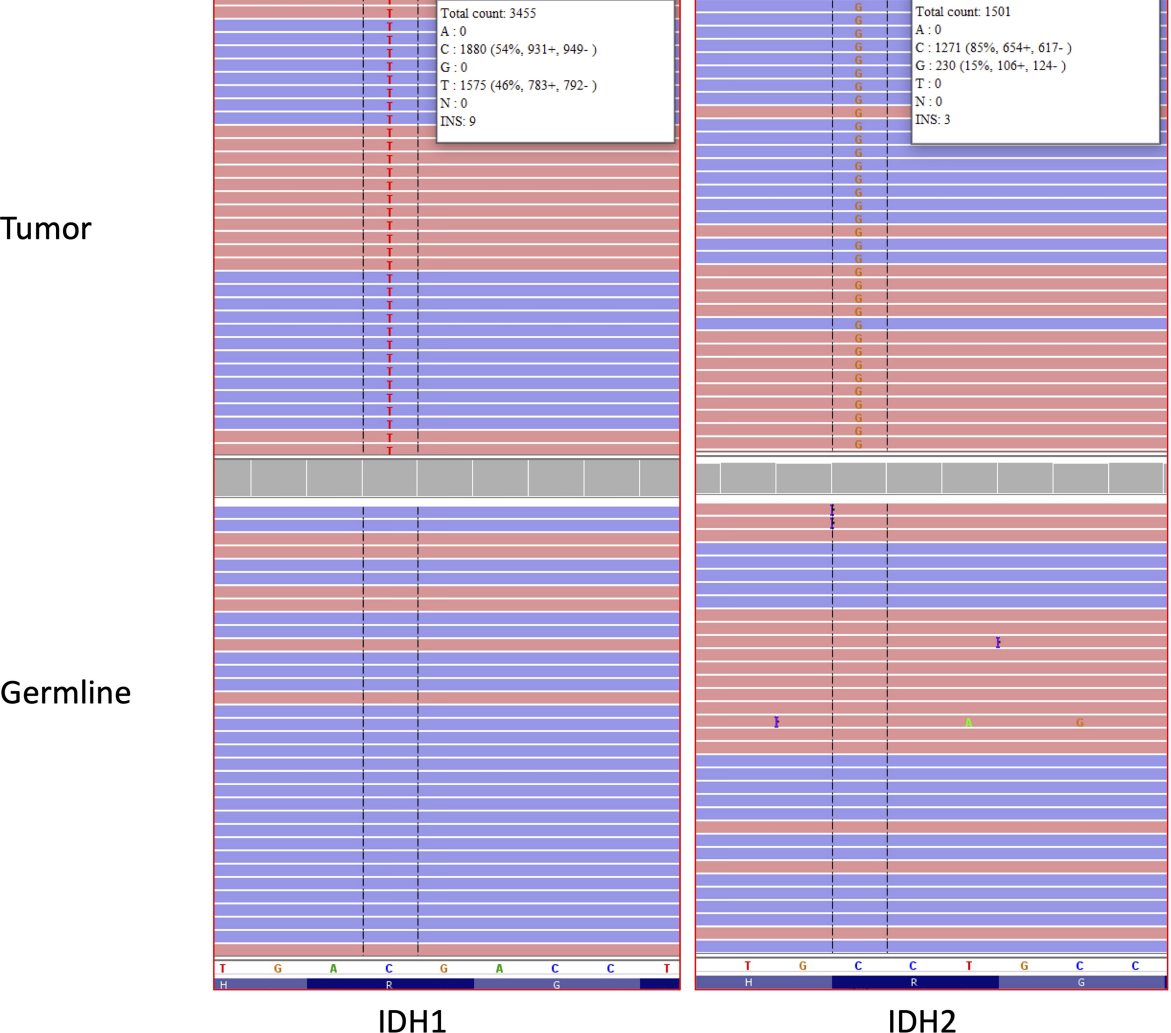
IGV traces showing somatic *IDH1* p.R132H (C>T nucleotide change) and *IDH2* p.R172S (C>G nucleotide change) mutations in the brain tumor tissue analyzed by NGS (Top). No *IDH1* or *IDH2* mutations were detected in DNA obtained from normal cells as a germline control (bottom). Red and blue bars represent forward and reverse sequencing reads, respectively. The reference nucleotide sequence for the depicted region of IDH1 and IDH2 is shown in the white bar in the lower portion of the figure. The “H”, “R” and “G” letters at the bottom of the figure represent the corresponding reference amino acid sequence for IDH1 and IDH2 (H = histidine, R= arginine, G=glycine).

**Figure 3:**
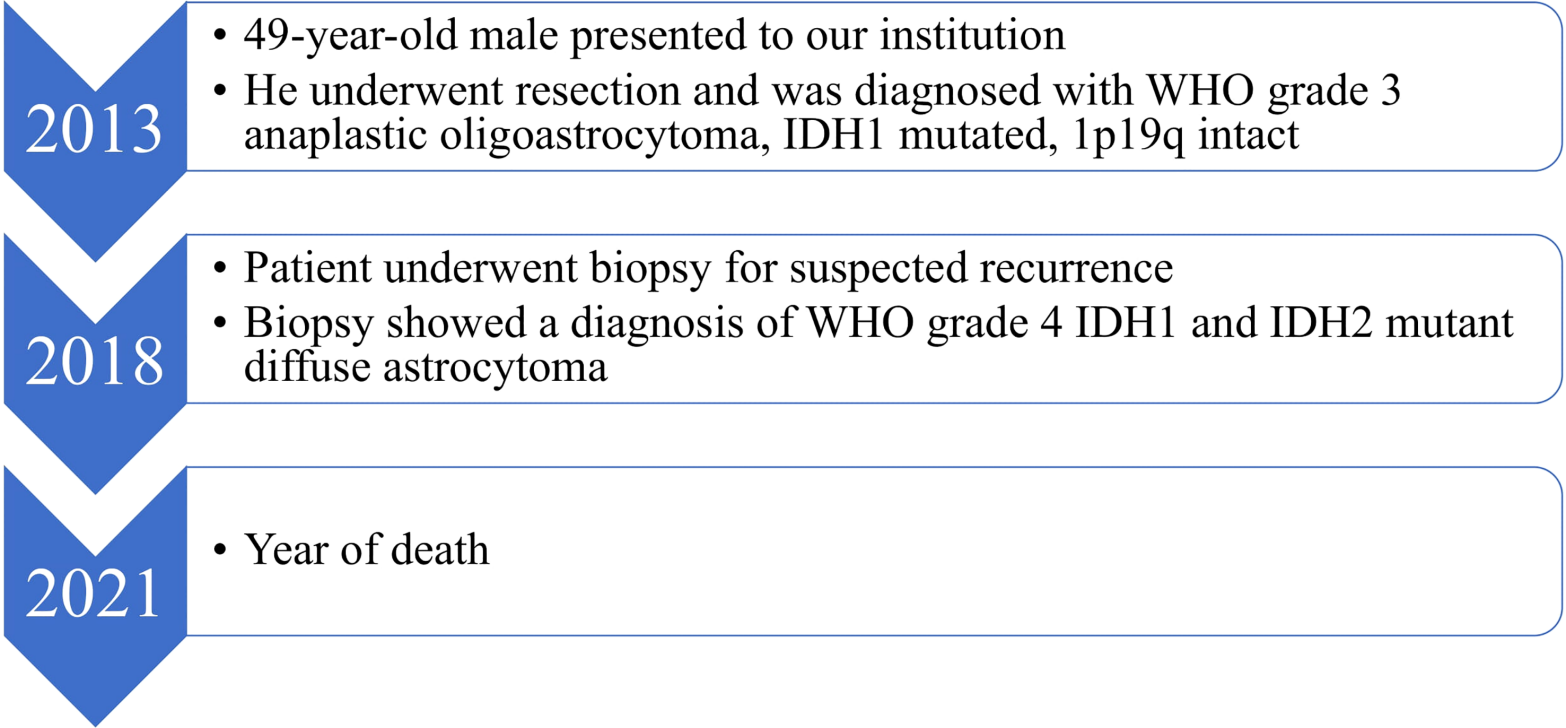
Patient care timeline from presentation to our institution until death.

A 49-year-old right-handed asymptomatic male presented to our institution in 2013 after magnetic resonance imaging (MRI) demonstrated an extensive lobular region of T2 signal hyperintensity within the right insula and perisylvian region. He initially underwent resection in 2007 and was diagnosed with grade 2 oligoastrocytoma, IDH1 mutant at an outside institution. The IDH1 mutation was determined by immunostaining. We do not have access to additional clinical information about the patient from that time since they were at an outside institution. He underwent a subtotal resection which revealed WHO grade 3 anaplastic oligoastrocytoma, IDH1 mutated, 1p19q intact. The 2013 diagnosis at our institution was determined by next generation sequencing (NGS) 50-gene panel showing IDH1 mutation (c.395G>A p.R132H), no IDH2 mutation, and TP53 mutation (c.817C>T p.R273C). ATRX was not included in the 50-gene panel, thus not tested. The patient then received 200mg/m^2^ per day temozolomide (TMZ) as chemotherapy for 8 cycles, followed by intensity-modulated radiation therapy (IMRT) to a total of 57 Gy in 30 fractions. In 2018, the patient underwent a surgical biopsy for suspected tumor progression that demonstrated WHO grade 4 *IDH1* and *IDH2* mutant diffuse astrocytoma. Microscopic examination of hematoxylin and eosin (H&E) stained sections of the biopsy showed a densely cellular, highly mitotic diffuse glioma with vascular proliferation composed predominantly of cells with relatively uniform round nuclei, many of which had perinuclear halos (**Figures 1A–E**). Mitotic activity was quantified at 8 mitoses per 10 high-power fields (HPF) on H&E stained sections, and at 17 mitoses per 10 HPF using phosphohistone H3 (pHH3). Computer-assisted automated quantification showed an elevated single field Ki67 antigen (MIB1) labeling index of 17.5%, with an average index of 10.3% over 4 hotspot fields. NGS identified the following somatic mutations: *IDH1* R132H, *IDH2* R172S, *TP53* R273C, and *ATRX* c3565del p.L1189* (**Figure 2**). The 2018 diagnosis at our institution was determined using a 126-gene NGS panel which also included ATRX. The IDH1 and TP53 mutations identified were identical to those identified in 2013. The patient subsequently underwent surgical resection and was then managed with radiation *via* volumetric modulated arc therapy (VMAT) of 40 Gy in 20 fractions followed by 150mg/m^2^ per day of TMZ chemotherapy for 8 cycles. He died in 2021 (**Figure 3**).

## 3 Discussion

Our case illustrates the complexity and variability regarding IDH mutations in gliomas. The normal role of *IDH1* and *IDH2* are to convert isocitrate to alpha-ketoglutarate. Mutations in *IDH1/IDH2* result in the conversion of alpha-ketoglutarate to 2-hydroxyglutarate, which functions as an oncometabolite and is a key driver of gliomagenesis (2, 8). Glioma-specific mutations in *IDH1* are known to most frequently affect codon 132, while the *IDH2* mutations typically affect codon 172 (2, 9). Both mutations are known to be heterozygous and somatic, leading to amino acid substitutions (2, 5, 10). IDH1 and IDH2 enzymatically function in separate subcellular compartments, with IDH1 in cytosol and IDH2 in mitochondria. It has been shown that under hypoxia IDH1 mutated cells have a decreased ability to induce reductive carboxylation and instead rely on oxidative mitochondrial metabolism (11). In contrast, IDH2 mutated cells have shown the ability to maintain reductive carboxylation in a hypoxic state. Therefore, tumor maintenance and oncogenicity may be impacted by hypoxia depending on the mutation status of IDH1 and IDH2, however our understanding of this is limited since the metabolic communication between mutant IDH1 and IDH2 is not yet understood (11). There is a paucity in the literature regarding the different types and frequencies of *IDH1/IDH2* mutations in gliomas. A search of the literature identified 4 cases of concurrent *IDH1/IDH2* mutations in gliomas similar to our case (12). An additional case of concurrent IDH1 and IDH2 mutations in gliomas was found in the literature (13). This case was a WHO grade 3 astrocytoma and noted to have a prolonged time to recurrence. This case is similar to the 4 cases published by Hartmann et al. in that all are histologically WHO grade 3 (12, 13). In comparison, our case of concurrent IDH1 and IDH2 mutations was histologically WHO grade 4. It could be possible to study survival or outcomes in association with the detection of concurrent IDH1 and IDH2 mutation in gliomas, however we have limited information at this time and thus are unable to theorize further. A query of The Cancer Genome Atlas (TCGA) database of lower grade gliomas and glioblastoma (n=794 samples) identified 2 cases of concurrent *IDH1/IDH2* mutations (tendency for mutual exclusivity, p<0.001).

IDH mutated gliomas are known to be a larger percentage of lower grade gliomas while comprise only a small minority of grade 4 gliomas in comparison to IDH wildtype gliomas (14). A more detailed and multi-institutional evaluation of this rare histopathological feature may have an impact on the development of future therapeutic approaches for patients with IDH-mutant gliomas. Although several targeted therapeutic options have been currently investigated for the management of tumors with *IDH1* and/or *IDH2* mutations, such as acute myeloid leukemias and cholangiocarcinomas, they have still a limited role for the treatment of gliomas (15). The co-presence of *IDH1* and *IDH2* mutations in patients with glioblastomas, as shown in our case, may suggest the occurrence of currently unknown epigenetic and/or molecular mechanisms whose discovery may likely lead to important advances in patient management and prognosis. As our understanding of concurrent IDH1 and IDH2 mutations in gliomas develops, this directly impacts clinical diagnostic testing standards. In current clinical practice, the immunohistochemical detection of IDH1 R132H in gliomas precludes the obligation to proceed to sequencing and any further information about IDH2 (16). Thus, it is likely that the current rate of concurrent IDH1 and IDH2 mutations in glioma is underestimated. This has further implications in assessing the effectiveness of novel therapeutics in development that target the IDH mutation, with further study necessary to understand the influence of concurrent IDH1 and IDH2 mutations on survival. In our case, both mutations were detected in the same tissue section. The lower VAF of the IDH2 mutation suggests the possibility that the IDH2 mutation is present only in a subset of the cells compared to the IDH1 mutation. At present, it is not possible for us to determine if the mutations were present in the same tumor cells or in distinct tumor cells within the same tissue section. In particular, the investigation of current inhibitors of IDH mutations, epigenetic therapies, and peptide vaccines may significantly benefit from the reports and analyses of these rare cases.

## 4 Conclusion

IDH mutations are key diagnostic and prognostic indicators in the management of gliomas. These mutations vary in frequency and type, with little epidemiological data in the literature. Concurrent *IDH1/IDH2* mutations are rare and require further study to better evaluate their impact on patients’ prognoses and their response to targeted therapies.

## Data availability statement

The original contributions presented in the study are included in the article, further inquiries can be directed to the corresponding author.

## Ethics statement

The studies involving human participants were reviewed and approved by University of Texas MD Anderson Cancer Center. The patients/participants provided their written informed consent to participate in this study. Written informed consent was obtained for the publication of this case report.

## Author contributions

All authors contributed to the manuscript writing and preparation of the figures. All authors contributed to the article and approved the submitted version.

## References

[1] Yan H Parsons DW Jin G McLendon R Rasheed BA Yuan W. IDH1 and IDH2 mutations in gliomas. N Engl J Med (2009) 360:765–73. doi: 10.1056/NEJMoa0808710 PMC2820383 19228619

[2] Picca A Berzero G Di Stefano AL Sanson M. The clinical use of IDH1 and IDH2 mutations in gliomas. Expert Rev Mol Diagn (2018) 18:1041–51. doi: 10.1080/14737159.2018.1548935 30427756

[3] Louis DN Perry A Reifenberger G von Deimling A Figarella-Branger D Cavenee WK. The 2016 world health organization classification of tumors of the central nervous system: a summary. Acta Neuropathol (2016) 131:803–20. doi: 10.1007/s00401-016-1545-1 27157931

[4] Louis DN Perry A Wesseling P Brat DJ Cree IA Figarella-Branger D. The 2021 WHO classification of tumors of the central nervous system: a summary. Neuro Oncol (2021) 23:1231–51. doi: 10.1093/neuonc/noab106 PMC8328013 34185076

[5] Horbinski C. What do we know about IDH1/2 mutations so far, and how do we use it? Acta Neuropathol (2013) 125:621–36. doi: 10.1007/s00401-013-1106-9 PMC3633675 23512379

[6] Labussière M Idbaih A Wang X-W Marie Y Boisselier B Falet C. All the 1p19q codeleted gliomas are mutated on IDH1 or IDH2. Neurology (2010) 74:1886–90. doi: 10.1212/WNL.0b013e3181e1cf3a 20427748

[7] Tabouret E Nguyen AT Dehais C Carpentier C Ducray F Idbaih A. Prognostic impact of the 2016 WHO classification of diffuse gliomas in the French POLA cohort. Acta Neuropathol (2016) 132:625–34. doi: 10.1007/s00401-016-1611-8 27573687

[8] Xu W Yang H Liu Y Yang Y Wang P Kim S-H. Oncometabolite 2-hydroxyglutarate is a competitive inhibitor of α-ketoglutarate-dependent dioxygenases. Cancer Cell (2011) 19:17–30. doi: 10.1016/j.ccr.2010.12.014 21251613 PMC3229304

[9] Cancer Genome Atlas Research Network Brat DJ Verhaak RGW Aldape KD Yung WKA Salama SR. Comprehensive, integrative genomic analysis of diffuse lower-grade gliomas. N Engl J Med (2015) 372:2481–98. doi: 10.1056/NEJMoa1402121 PMC4530011 26061751

[10] Parsons DW Jones S Zhang X Lin JC-H Leary RJ Angenendt P. An integrated genomic analysis of human glioblastoma multiforme. Science (2008) 321:1807–12. doi: 10.1126/science.1164382 PMC2820389 18772396

[11] Grassian AR Parker SJ Davidson SM Divakaruni AS Green CR Zhang X. IDH1 mutations alter citric acid cycle metabolism and increase dependence on oxidative mitochondrial metabolism. Cancer Res (2014) 74:3317–31. doi: 10.1158/0008-5472.CAN-14-0772-T PMC4885639 24755473

[12] Hartmann C Meyer J Balss J Capper D Mueller W Christians A. Type and frequency of IDH1 and IDH2 mutations are related to astrocytic and oligodendroglial differentiation and age: a study of 1,010 diffuse gliomas. Acta Neuropathol (2009) 118:469–74. doi: 10.1007/s00401-009-0561-9 19554337

[13] Yuile A Satgunaseelan L Wei J Kastelan M Back MF Lee M. Implications of concurrent IDH1 and IDH2 mutations on survival in glioma–a case report and systematic review. Curr Issues Mol Biol (2022) 44:5117–25. doi: 10.3390/cimb44100348 PMC9600580 36286062

[14] Neff C Cioffi G Waite K Kruchko C Barnholtz-Sloan JS Ostrom QT. Molecular marker testing and reporting completeness for adult-type diffuse gliomas in the united states. Neuro-Oncology Pract (2023) 10:24–33. doi: 10.1093/nop/npac079 PMC9837780 36659967

[15] Pirozzi CJ Yan H. The implications of IDH mutations for cancer development and therapy. Nat Rev Clin Oncol (2021) 18:645–61. doi: 10.1038/s41571-021-00521-0 34131315

[16] Brat DJ Aldape K Bridge JA Canoll P Colman H Hameed MR. Molecular biomarker testing for the diagnosis of diffuse gliomas. Arch Pathol Lab Med (2022) 146:547–74. doi: 10.5858/arpa.2021-0295-CP PMC9311267 35175291

